# Mass Compression from Recurrent Lymphoma Mimicking Lower Extremity Cellulitis

**DOI:** 10.7759/cureus.2466

**Published:** 2018-04-12

**Authors:** David G Li, Katherine M Krajewski, Arash Mostaghimi

**Affiliations:** 1 Department of Dermatology, Brigham & Women’s Hospital, Harvard Medical School, Boston, USA; 2 Department of Imaging, Dana-Farber Cancer Institute; Department of Radiology, Brigham and Women’s Hospital, Harvard Medical School, Boston, USA

**Keywords:** cellulitis, pseudocellulitis, lymphoma, compression, diffuse large b-cell lymphoma (dlbcl), cancer, erythema, edema

## Abstract

Cellulitis is a common skin and soft tissue infection with substantial misdiagnosis rates due to its nonspecific clinical characteristics. In this report, we present a patient with recurrent metastatic diffuse large B-cell lymphoma (DLBCL) masquerading as a unilateral lower extremity cellulitis. A 62-year-old man with a history of DLBCL, in remission for two years, presented with a two-week history of swelling and erythema of the right thigh and leg. Despite treatment with clindamycin and cephalexin, the redness and swelling continued to progress. On presentation to the emergency department, vitals were within normal limits, laboratory workup was significant only for borderline anemia and thrombocytopenia, and bilateral lower extremity ultrasound was negative for a clot. The patient was evaluated by a dermatologist who recommended further imaging workup for proximal vascular compression given the uniformity of inflammation and edema in the absence of fever or systemic symptoms. Imaging revealed retroperitoneal lymphadenopathy, right pelvic side wall and right inguinal lymphadenopathy, an intramuscular lymphomatous involvement of the right iliopsoas muscle, and mass compression of the right external iliac vein. Bone marrow and soft-tissue biopsies confirmed recurrent and metastatic DLBCL. In this patient, the atypical cellulitis-like features are likely due to venous and lymphatic obstruction secondary to mass effect from metastasis. Going forward, clinicians should consider compression-induced edema as a sign of primary or recurrent malignancy in patients with refractory or atypical cellulitis.

## Introduction

Cellulitis is an infection of the deep dermis and subcutaneous tissue that often presents as expanding erythema, swelling, warmth, and tenderness of the affected site [1]. The lack of a “gold standard” diagnostic modality and nonspecific clinical characteristics results in frequent misdiagnosis of cellulitis mimickers (pseudocellulitis) as cellulitis, leading to inappropriate antibiotic therapy and potential complications [2]. In this report, we present a case of recurrent metastatic diffuse large B-cell lymphoma (DLBCL) mimicking a unilateral lower extremity cellulitis.

## Case presentation

A 62-year-old man with a history of DLBCL previously treated with autologous stem cell transplant and combination chemotherapy with rituximab, in remission for two years, presented with a two-week history of swelling and erythema of the right thigh and leg. The symptoms began acutely as an erythematous patch on his right thigh, at which time he was started on oral clindamycin for presumed cellulitis. Despite a seven-day-course of clindamycin, the patient reported progression of erythema down his leg and was subsequently transitioned to three days of oral cephalexin, also without improvement.

**Figure 1:**
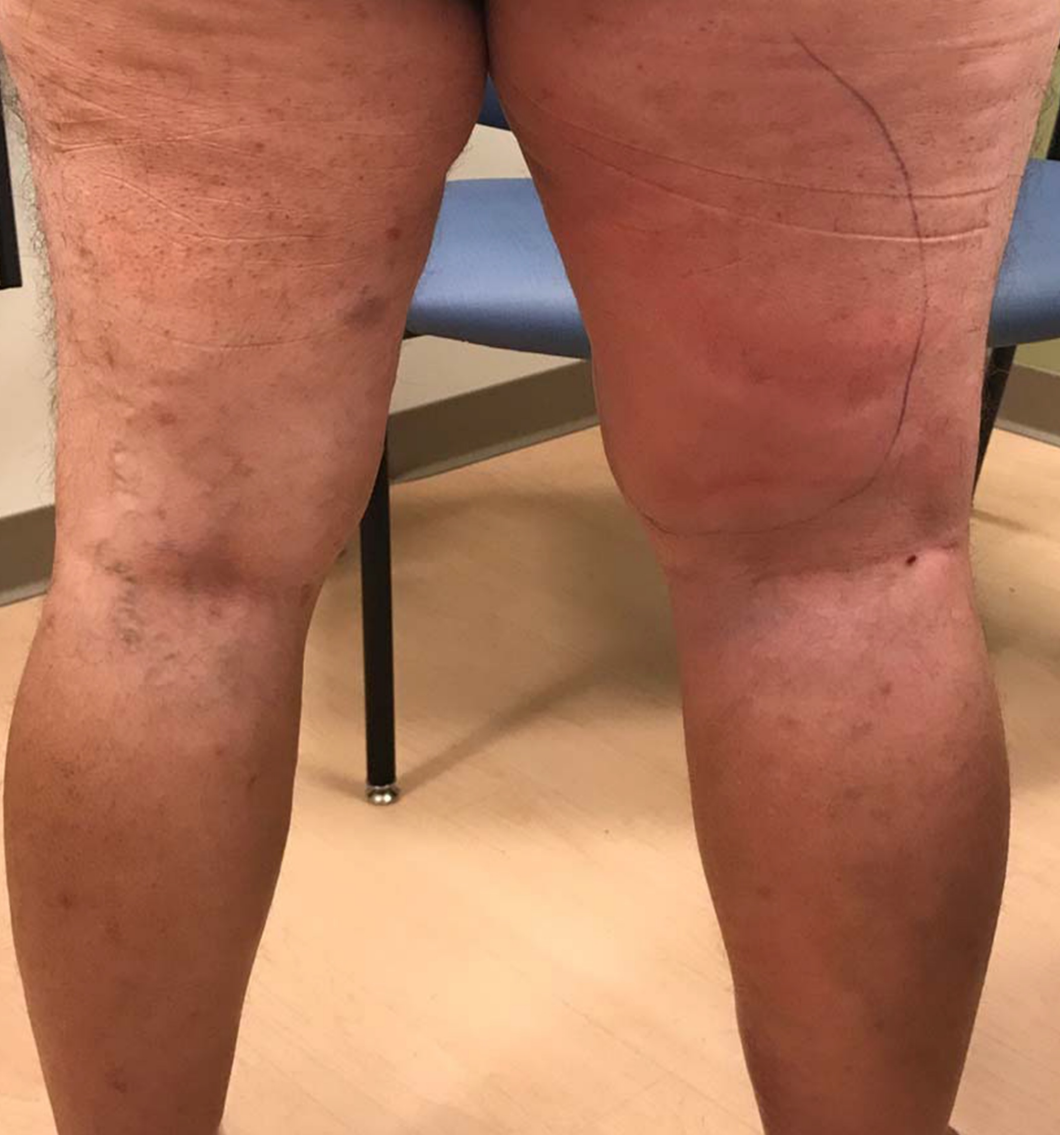
Compression lymphadenopathy mimicking cellulitis One confluent and edematous plaque over the right posterior thigh.

**Figure 2:**
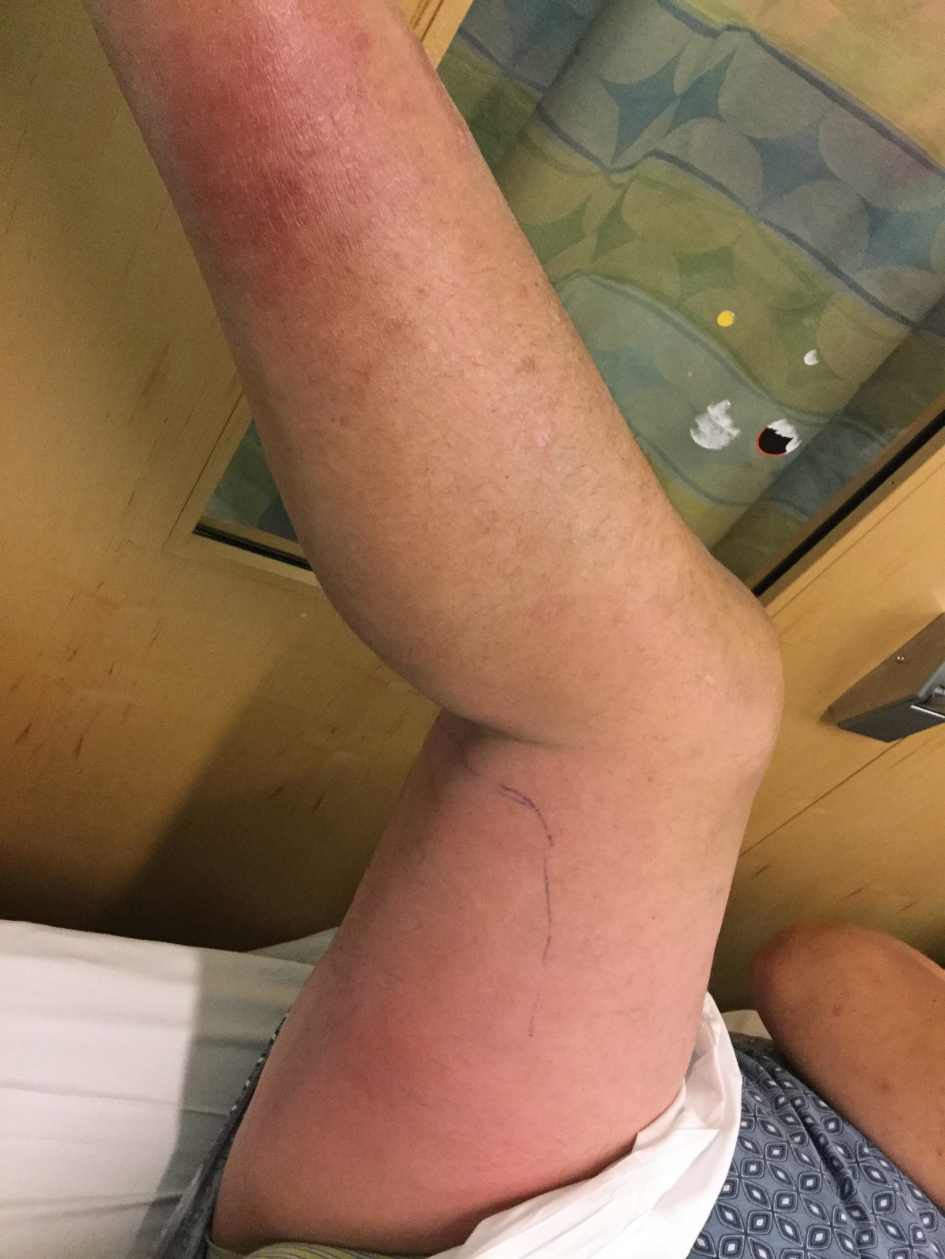
Compression lymphadenopathy mimicking cellulitis ​​​​​​​Poorly demarcated erythematous and edematous plaques over the right posteromedial leg and right posterior thigh extending to the right buttocks.

On presentation, the patient had confluent erythema and mild edema from the right upper thigh to the medial calf without tenderness or pruritus (Figures 1-2). His blood pressure was 123/62 mmHg; heart rate 83/minute; temperature 37.0 C (98.6 F); respiratory rate 18; and oxygen saturation 97%. Complete blood count was remarkable only for borderline anemia and thrombocytopenia. The patient’s asymmetry, leukocytosis, tachycardia, age ≥ 70 (ALT-70) lower extremity cellulitis score was three (unilateral disease, no leukocytosis or tachycardia, age < 70), an indeterminate finding that may benefit from further workup or consultation [3]. Ultrasound evaluation of the lower extremities for clot was unremarkable. The patient was evaluated by dermatology during this encounter, who recommended imaging workup for possible proximate vascular compression considering the uniformity of inflammation and edema in absence of fever or systemic symptoms.

**Figure 3:**
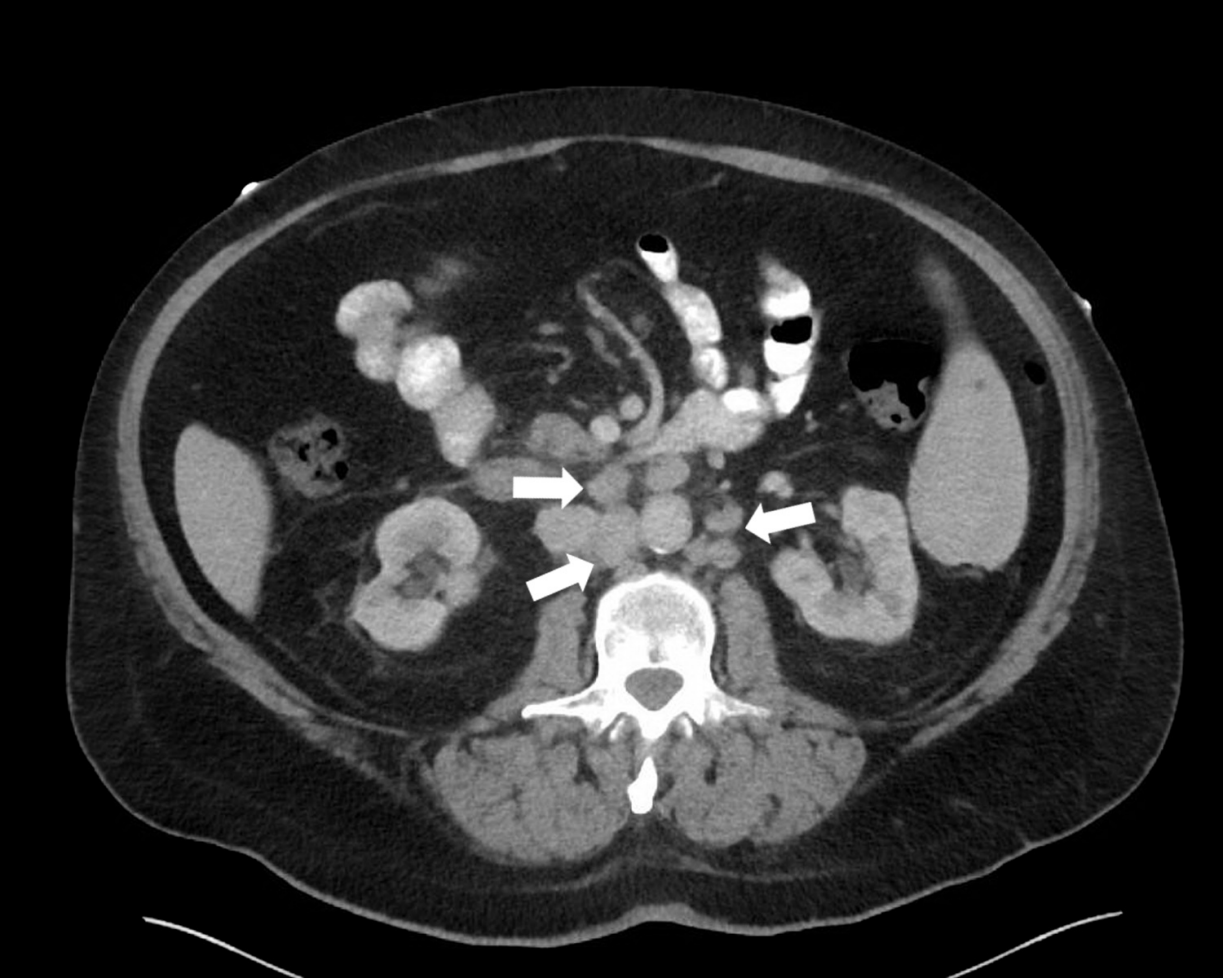
Recurrent diffuse large B-cell lymphoma Computed tomography (CT) demonstrating retroperitoneal adenopathy (arrows).

**Figure 4:**
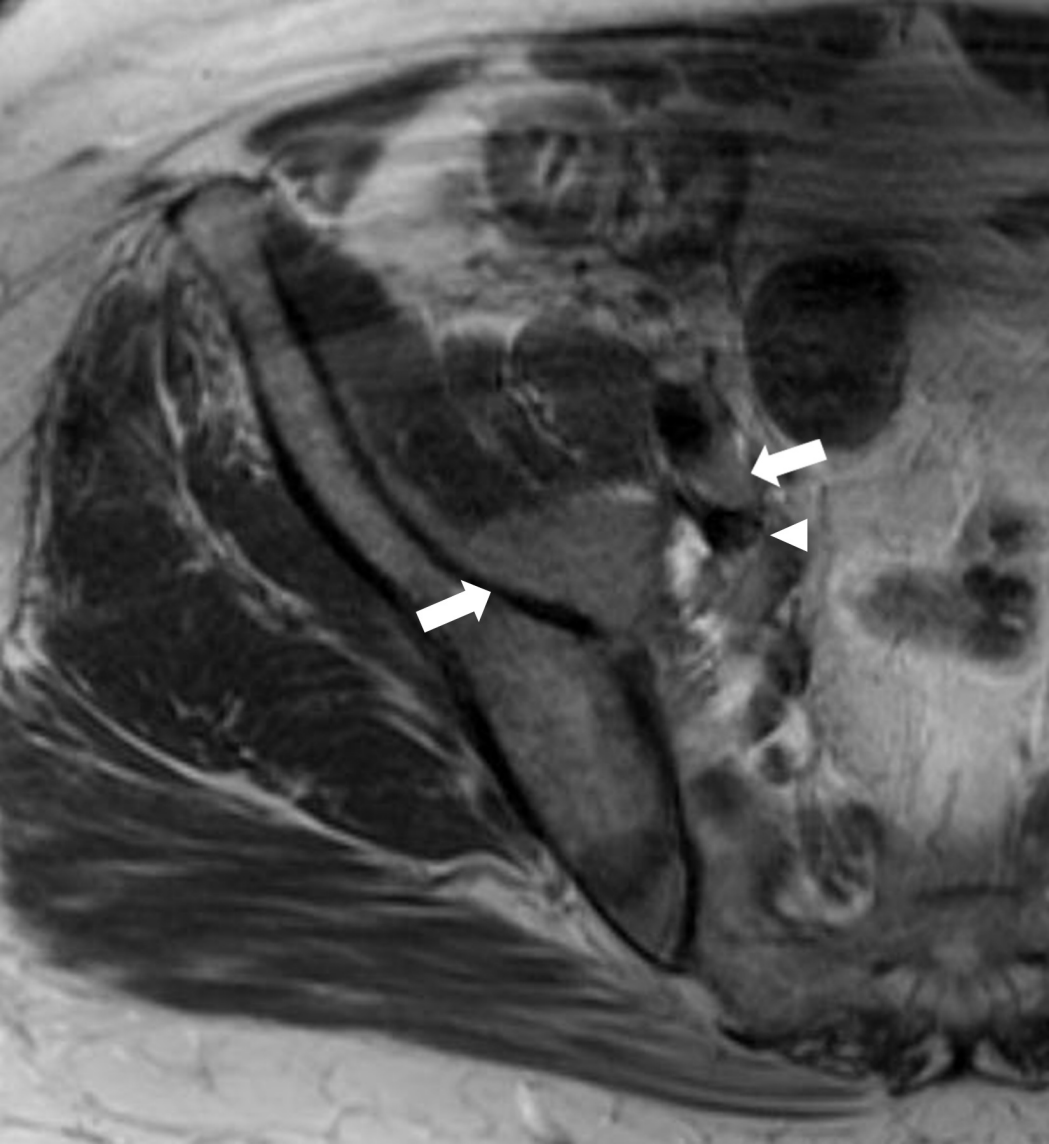
Recurrent diffuse large B-cell lymphoma Magnetic resonance imaging (MRI) revealing hyperintense lymphoma lesions involving the right iliopsoas muscle (left sided arrow), right external iliac node (right sided arrow) with subsequent compression of the right external iliac vein (arrowhead).

Computed tomography (CT) of the abdomen and pelvis was performed and was remarkable for retroperitoneal lymphadenopathy (Figure 3). Magnetic resonance imaging (MRI) showed lymphadenopathy of the right pelvic side wall and right inguinal regions, intramuscular lymphomatous involvement of the right iliopsoas muscle, and mass compression of the right external iliac vein (Figure 4). Antibiotics were discontinued and the patient improved with leg elevation. Bone marrow and soft-tissue biopsies (skin biopsy not performed) after discharge confirmed recurrent and metastatic DLBCL.

## Discussion

A subset of diseases resembling cellulitis (aka pseudocellulitis) has been previously documented in the context of malignancy, including carcinoma erysipeloides (cutaneous metastasis of visceral carcinomas such as pulmonary or prostate adenocarcinoma) presenting as erythematous, infiltrative patches, and plaques with local inflammation [4-5]. Cellulitis-like Sweets Syndrome, a neutrophilic dermatosis, has been documented to precede the discovery of a malignant neoplasm including myeloproliferative disease [6]. Bone metastasis in patients with underlying malignancy have also manifested as pseudocellulitis, leading to delays in care and unnecessary antimicrobial therapy [7].

Acute compression secondary to malignancy, as described in the present case, should also be added to the differential for pseudocellulitis. Increased awareness of cutaneous pseudocellulitis as a manifestation of underlying malignancy, especially in high-risk patients (e.g., patients at risk for recurrence, with family history of cancer, or past exposure to carcinogens) [8], is important not only to avoid unnecessary antibiotics and hospitalization, but also to begin appropriate and timely therapy for malignancy.

While there have also been reports of DLBCL mimicking cellulitis [9-10], the present case differs in the mechanism responsible for pseudocellulitis features. In our patient, the atypical cellulitis-like features are likely the result of venous and lymphatic obstruction (involving the right external iliac vein and inguinal/pelvic side wall lymph nodes, respectively) secondary to mass effect from metastasis. Although the patient presented with features suggestive of cellulitis, there was a progression of symptoms despite a ten-day course of two different oral antibiotics. Although the lack of response to multiple antibiotics decreases the likelihood of cellulitis, it does not eliminate the possibility of resistant or atypical microorganisms. However, the patient denied fevers or chills, was hemodynamically stable, had no leukocytosis, and had an ALT-70 score of three, which was indeterminate and would likely benefit from specialty consultation [3].

## Conclusions

In this patient who did not respond to a standard cellulitis treatment protocol, the atypical cellulitis-like features are likely due to venous and lymphatic obstruction secondary to mass effect from metastasis, as seen from clinical and radiological evidence. Going forward, clinicians should consider compression-induced edema as a sign of primary or recurrent malignancy in patients with refractory or atypical cellulitis.
